# Multidrug therapy does not eliminate the risk of ocular disability in leprosy

**DOI:** 10.1371/journal.pntd.0014859

**Published:** 2026-11-06

**Authors:** Matthew Willis

**Affiliations:** 1 Department of Global Health, Institute of Public Health and Nursing Research, University of Bremen, Bremen, Germany; 2 Centre for Public Health, School of Medicine, Dentistry and Biomedical Sciences, Queen’s University Belfast, Belfast, United Kingdom; Marie Adelaide Leprosy Center, PAKISTAN

## Abstract

Whilst multidrug therapy can provide a microbiological cure for leprosy infection, it does not reverse nerve damage that is already established, nor remove the risk of the occurrence of future ocular complications. Ocular conditions associated with leprosy such as lagophthalmos, reduced corneal sensation, exposure keratopathy, uveitis and other sight and eye threatening conditions may be present at diagnosis, develop during treatment, or even progress long after treatment has already been completed. Existing guidance already recommends eye assessment during treatment, but continuity after treatment varies between settings. This viewpoint therefore argues for implementing these recommendations through a minimum pathway which links assessment during treatment to risk-stratified follow-up after treatment completion, clear referral responsibilities, supported self-care and monitoring, and routine assessment of visual outcomes in paper or electronic records. To be successful these measures should be integrated within existing leprosy, primary-care and eye-care services, with links to non-communicable disease programmes where these can support long-term care. Achieving zero disability due to leprosy requires recognising that treatment completion is not the end of ocular risk.

## Author summary

Leprosy can damage the nerves responsible for eyelid closure and corneal sensation. This can leave the eye exposed to dryness, injury and infection, and the reduced sensation may prevent the person affected from recognising that damage is occurring. These risks do not necessarily end when multidrug therapy is completed. Although eye examinations are already recommended during treatment, people affected by leprosy may have difficulty accessing continued eye care afterwards. This viewpoint therefore makes the case for linking these assessments to a clear plan for continued care. Those with lagophthalmos, reduced corneal sensation, previous eye disease or other risk factors should continue to receive regular follow-up even after treatment completion. Leprosy programmes should also record whether referrals are completed and whether vision is maintained. Links with existing eye-health and non-communicable disease programmes could support lifelong care where needed, including for disabilities caused by other neglected tropical diseases. These would help ensure that people released from treatment are not subsequently lost to vital sight-preserving care.

## Multidrug therapy does not prevent all future disability

Leprosy is a chronic infectious neglected tropical disease which primarily affects the skin and peripheral nerves, and can lead to ocular morbidity and disability [1–3]. Multidrug therapy (MDT) interrupts transmission of the causative bacteria and provides a microbiological cure. However, much of the long-term disability that is associated with leprosy results from inflammatory complications and nerve damage which may already be established by the time treatment begins, and also from leprosy reactions which can occur before, during, or after treatment [1,2]. Completion of MDT therefore does not end the biological risk to sight in those affected by leprosy, even where follow-up is available [2].

This is particularly relevant to the eye and to sight. Facial nerve involvement in those affected by leprosy can impair blinking and eyelid closure, resulting in lagophthalmos and chronic exposure of the cornea. Trigeminal nerve involvement may compound this further by reducing corneal sensation, meaning that dryness, injury or early keratitis may not be recognised by the person affected [3,4]. These mechanisms in many cases occur together, leaving the cornea both unprotected and unable to produce an effective warning response in the form of pain. Uveitis and other inflammatory complications may cause further structural damage to the eye, while cataract, glaucoma and refractive error can all add to the overall burden of visual impairment [3,4].

Ocular disease in those affected by leprosy may be present at the time of diagnosis, develop during MDT or occur after treatment has already been completed. In a prospective cohort of people with multibacillary leprosy followed after MDT, new leprosy-related ocular disease and potentially blinding ocular conditions occurred at rates of 5.65 and 3.86 per 100 patient-years, respectively [5]. Studies of treated populations in both India and the United Kingdom have similarly identified substantial ocular morbidity, including lagophthalmos, corneal disease, uveitis and glaucoma [6,7]. It has also been identified that sight-threatening lesions are still present among those who have already completed MDT [8]. Although geographically limited and often based on specialist clinics, this evidence demonstrates continuing biological risk rather than the absence of follow-up in every programme.

Visual impairment may also worsen the existing functional consequences of leprosy among people already living with sensory loss, impaired mobility or a visible deformity [9]. The ocular consequences of leprosy should therefore not be considered separately from its wider effects on both individual independence and daily function.

## The gap after treatment completion

The WHO Global Leprosy Strategy 2021–2030 places the management of complications and prevention of new disability among its four key strategic pillars [1]. Existing guidance includes eye assessment during MDT, disability recording, self-care and referral. The gap is therefore between these recommendations and their consistent implementation, including after treatment completion. Evidence from Brazil has shown that physical disability can worsen many years after release from treatment, particularly among people who experienced complications during their MDT courses [10].

Some programmes, including those supported by NGOs, already provide post-treatment follow-up and rehabilitation. Elsewhere, the responsibility for continued ocular care can become unclear. Specialist leprosy services may have limited capacity for prolonged follow-up, while general eye-care services may have limited experience of recognising and managing the specific ocular complications of leprosy. People affected by leprosy may therefore fall between disease-control programmes, primary care and specialist eye-health centres.

Disability grading should support rather than replace clinical examination. Earlier WHO definitions include eye problems without severe visual impairment as grade 1 and lagophthalmos, iridocyclitis or corneal opacity as grade 2 [11]. A subsequent consensus exercise retained grade 1 for absent spontaneous blinking without visible eye damage and recommended specialist referral [12]. Current national instructions should be used when completing disability grades and the Eye-Hand-Foot (EHF) score [12]. Reduced corneal sensation, early exposure keratopathy, unilateral visual loss and changes in eyelid function may all require assessment before severe visual impairment develops [3,9].

## A minimum pathway for continued ocular care

A minimum pathway for continued eye care should build on the Leprosy Examination and Treatment Card or its national equivalent, including the WHO model patient card [2]. The proposed pathway links existing assessments to risk-based follow-up, supported self-care, referral and outcome measurement beyond MDT completion (Fig 1).

**Fig 1:**
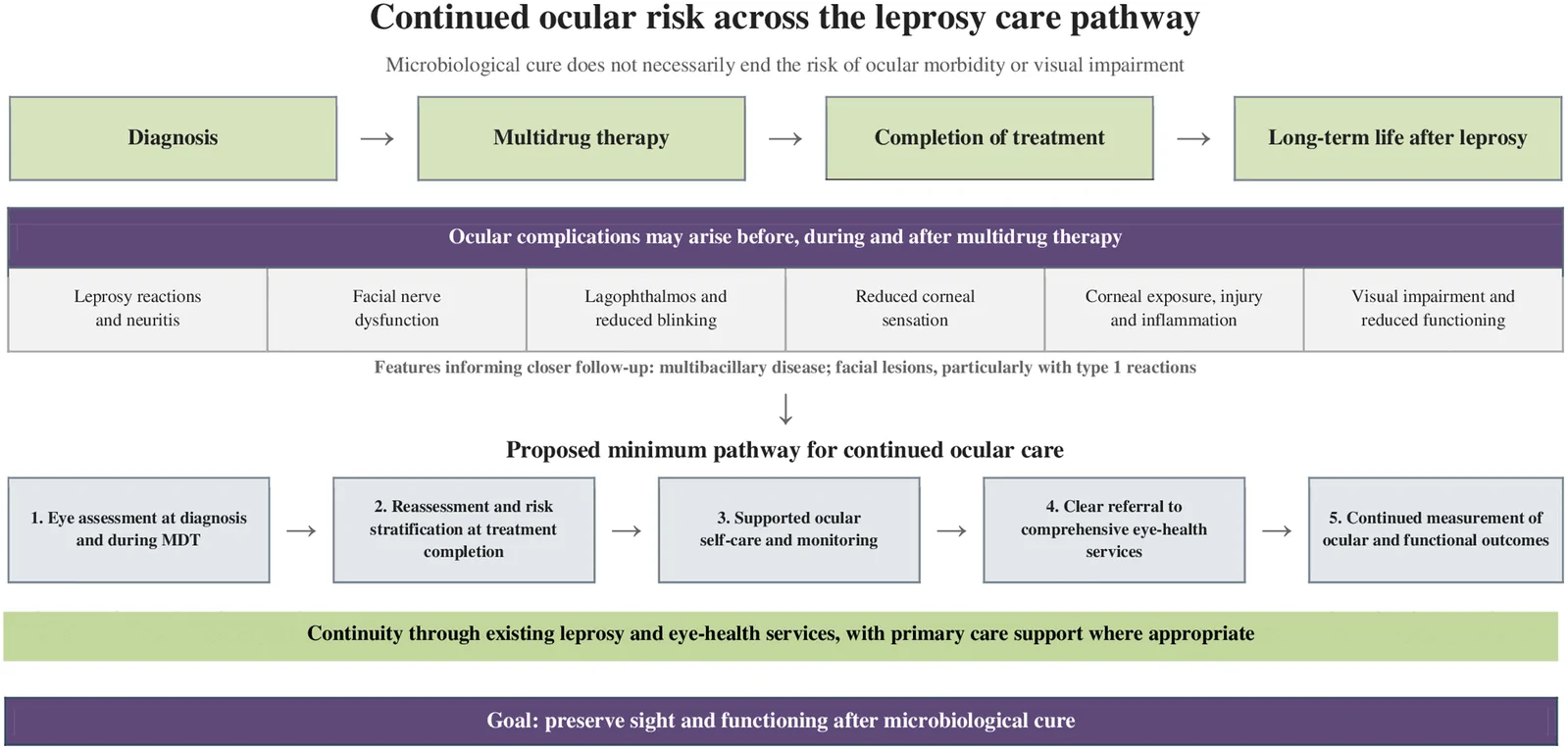
Continued ocular risk across the leprosy care pathway: Ocular complications may arise before, during and after MDT. The proposed pathway links existing assessments and disability recording to continued care through leprosy, eye-health and non-communicable disease services.

A basic eye exam should be completed when leprosy is diagnosed, regularly during MDT according to national guidance, and at treatment completion [2,12]. At minimum, this should document presenting visual acuity in each eye, eyelid closure, blink function, visible eyelid deformity, corneal clarity and symptoms such as redness, photophobia, discharge or deterioration in vision [3,9]. Findings, including disability grades, should be recorded on the existing paper or electronic patient card. Training non-specialist leprosy workers is feasible [13], but does not replace specialist assessment. Training, supervision, and access to ophthalmologists are needed so that recording of a grade does not substitute for an adequate examination.

The ocular risk profile should be reassessed when MDT is completed. Multibacillary disease, facial lesions (particularly with Type 1 reactions), and existing ocular damage should inform closer follow-up [3,5,14]. People with lagophthalmos, facial nerve dysfunction, reduced corneal sensation, previous ocular inflammation, recurrent reactions or visual impairment require continued ophthalmic follow-up [3,5–7]. The ideal duration and frequency of post-treatment ophthalmic follow-up have not been adequately established. Nonetheless, a lack of consensus should not result in discharge without a named service, review date and route for urgent reassessment. New visual loss, a red or photophobic eye, corneal injury, or new or incomplete eyelid closure warrants prompt ophthalmic assessment.

People affected by leprosy and, where appropriate, their family members should also receive practical education on eye self-care and monitoring. People require education that reduced corneal sensation means that the lack of pain cannot be considered reassuring [3,9]. Previously published evidence suggests that knowledge and practice of eye care among people affected by leprosy is in many cases limited and that family members often contribute to their care [15]. Education should be tailored to the local population, repeated alongside basic self-care and linked to an accessible referral pathway, rather than shifting responsibility for preventable visual loss entirely onto the individual affected.

Referral should not be restricted to disease directly attributable to leprosy. People affected by leprosy may also require cataract surgery, refractive correction, glaucoma treatment or low-vision support. Corticosteroids which are used to manage reactions can also contribute to cataract and glaucoma; which although not entirely attributable to leprosy infection, may be more likely among those affected by leprosy [3,6,9]. Leprosy services should therefore provide an entry point into affordable community eye-care, particularly in underserved communities.

## Measuring whether sight is maintained

Indicators reported by leprosy programmes largely focus on case detection, completion of MDT and the grade of disability at diagnosis [1,11]. A minimum set of ocular outcomes could additionally comprise visual acuity in each eye, lagophthalmos or corneal disease, new ocular complications, referral date, referral completion and treatment received. In paper-based services, these could be added to the existing card and a simple referral register, with a named worker checking the outstanding referrals. Where electronic records exist, the same fields could support recall and referral tracking. Not all measures need national reporting; services could review missed follow-up, completed referrals and change in vision locally.

Where national eye-health programmes exist, leprosy services should agree referral and information sharing agreements with them. Non-communicable disease programmes could support continuing appointments and rehabilitation for chronic disabilities, including those caused by other neglected tropical diseases, with specialist eye-care services retained. Where leprosy services remain the main provider, they should retain follow-up responsibility and establish referral links with tertiary ophthalmology services or outreach services.

## Conclusion

The introduction of MDT has transformed the treatment and control of leprosy; however, completion of MDT does not reverse already established nerve damage nor remove the possibility of future ocular complications. The contribution of this viewpoint is to advocate for the linking of existing assessments during MDT to risk-stratified follow-up afterwards, with clear clinical responsibility and ocular outcomes recorded in paper or electronic systems. Supported self-care and accessible referrals remain essential.

Importantly, this viewpoint argues that these measures should be integrated within existing leprosy and eye-health programmes, with non-communicable disease services supporting long-term care where feasible. Ultimately, the ambition of zero disability due to leprosy cannot be achieved through MDT alone. Completion of MDT must remain an important stage in care, but should not mark the end of efforts to preserve sight.

## Abbreviations

EHF: Eye-Hand-Foot
MDT: Multidrug therapy

## References

[1] World Health Organization. Towards zero leprosy: Global leprosy (Hansen’s Disease) strategy 2021–2030. 2021.

[2] Leprosy/Hansen Disease: Management of reactions and prevention of disabilities. [cited in 2026 Oct 15]. https://www.who.int/publications/i/item/9789290227595

[3] Grzybowski A, Nita M, Virmond M. Ocular leprosy. Clin Dermatol. 2015;33(1):79–89. doi: 10.1016/j.clindermatol.2014.07.003 25432813

[4] Pavezzi PD, do Prado RB, Boin Filho PÂ, Gon ADS, Tuma B, Fornazieri MA, et al. Evaluation of ocular involvement in patients with Hansen’s disease. PLoS Negl Trop Dis. 2020;14(9):e0008585. doi: 10.1371/journal.pntd.0008585 32956360 PMC7505469

[5] Daniel E, Ffytche TJ, Kempen JH, Rao PSSS, Diener-West M, Courtright P. Incidence of ocular complications in patients with multibacillary leprosy after completion of a 2 year course of multidrug therapy. Br J Ophthalmol. 2006;90(8):949–54. doi: 10.1136/bjo.2006.094870 16707521 PMC1857220

[6] Parikh R, Thomas S, Muliyil J, Parikh S, Thomas R. Ocular manifestation in treated multibacillary Hansen’s disease. Ophthalmology. 2009;116(11):2051-7.e1. doi: 10.1016/j.ophtha.2009.04.021 19766316

[7] Malik ANJ, Morris RW, Ffytche TJ. The prevalence of ocular complications in leprosy patients seen in the United Kingdom over a period of 21 years. Eye (Lond). 2011;25(6):740–5. doi: 10.1038/eye.2011.43 21423140 PMC3178125

[8] Ffytche TJ. Residual sight-threatening lesions in leprosy patients completing multidrug therapy and sulphone monotherapy. Lepr Rev. 1991;62(1):35–43. doi: 10.5935/0305-7518.19910005 2034023

[9] Hogeweg M, Keunen JEE. Prevention of blindness in leprosy and the role of the Vision 2020 Programme. Eye (Lond). 2005;19(10):1099–105. doi: 10.1038/sj.eye.6701984 16304590

[10] Dos Santos AR, Silva PRS, Steinmann P, Ignotti E. Disability progression among leprosy patients released from treatment: a survival analysis. Infect Dis Poverty. 2020;9(1):53. doi: 10.1186/s40249-020-00669-4 32448360 PMC7245886

[11] Brandsma JW, Van Brakel WH. WHO disability grading: operational definitions. Lepr Rev. 2003;74(4):366–73. doi: 10.47276/lr.74.4.366 14750582

[12] Cross H. A Delphi exercise to refine the WHO three-point disability grading system for leprosy, and to develop guidelines to promote greater accuracy and reliability of WHO disability recording. Lepr Rev. 2014;85(1):18–28. doi: 10.47276/lr.85.1.18 24974439

[13] Courtright P, Lee HS, Lewallen S. Training for primary eye care in leprosy. Bull World Health Organ. 1990;68(3):347–51. 2393981 PMC2393073

[14] Hogeweg M, Kiran KU, Suneetha S. The significance of facial patches and type I reaction for the development of facial nerve damage in leprosy. A retrospective study among 1226 paucibacillary leprosy patients. Lepr Rev. 1991;62(2):143–9. doi: 10.5935/0305-7518.19910016 1870376

[15] Yowan P, Danneman K, Koshy S, Richard J, Daniel E. Knowledge and practice of eye-care among leprosy patients. Indian J Lepr. 2002;74(2):129–35. 12708731

